# Wounding response in Porifera (sponges) activates ancestral signaling cascades involved in animal healing, regeneration, and cancer

**DOI:** 10.1038/s41598-022-05230-x

**Published:** 2022-01-25

**Authors:** Yu-Chen Wu, Soeren Franzenburg, Marta Ribes, Lucía Pita

**Affiliations:** 1 Research Unit Marine Microbiology, Department Marine Ecology, GEOMAR Helmholtz Centre for Ocean Research, Kiel, Germany; 2 Christian-Albrechts University of Kiel, Kiel, Germany; 3 Institute of Clinical Molecular Biology (IKMB), Christian-Albrechts University of Kiel, Kiel, Germany; 4 Present Address: Department Marine Biology and Oceanography, Institute of Marine Sciences (ICM-CSIC), Barcelona, Spain

**Keywords:** Molecular ecology, Marine biology

## Abstract

Upon injury, the homeostatic balance that ensures tissue function is disrupted. Wound-induced signaling triggers the recovery of tissue integrity and offers a context to understand the molecular mechanisms for restoring tissue homeostasis upon disturbances. Marine sessile animals are particularly vulnerable to chronic wounds caused by grazers that can compromise prey’s health. Yet, in comparison to other stressors like warming or acidification, we know little on how marine animals respond to grazing. Marine sponges (Phylum Porifera) are among the earliest-diverging animals and play key roles in the ecosystem; but they remain largely understudied. Here, we investigated the transcriptomic responses to injury caused by a specialist spongivorous opisthobranch (i.e., grazing treatment) or by clipping with a scalpel (i.e., mechanical damage treatment), in comparison to control sponges. We collected samples 3 h, 1 d, and 6 d post-treatment for differential gene expression analysis on RNA-seq data. Both grazing and mechanical damage activated a similar transcriptomic response, including a clotting-like cascade (e.g., with genes annotated as transglutaminases, metalloproteases, and integrins), calcium signaling, and Wnt and mitogen-activated protein kinase signaling pathways. Wound-induced gene expression signature in sponges resembles the initial steps of whole-body regeneration in other animals. Also, the set of genes responding to wounding in sponges included putative orthologs of cancer-related human genes. Further insights can be gained from taking sponge wound healing as an experimental system to understand how ancient genes and regulatory networks determine healthy animal tissues.

## Introduction

Tissue homeostasis is the capacity to maintain, via feedback loops, the internal conditions that allow the proper functioning of an animal. If the mechanisms of homeostasis fail, diseases like chronic wounds or cancer will develop^1–6^. In fact, there are intriguing parallels between wound healing and cancer^3–6^. Wound healing activates mechanisms that are strikingly similar to those that, when dysregulated, trigger tumor initiation and progression^3–6^. Processes like inflammation, activation of migration, or enhanced proliferation, regulated in wound healing and uncontrolled in cancer^6^, are largely regulated by ancient genes that evolved at the emergence of multicellularity^7^. Thus, an evolutionary perspective into the molecular mechanisms of homeostasis can reveal conserved gene interactions and functions in coordinating cellular behavior and sensing the environment. Wounding response and healing research offers a context to understand fundamental processes that control how healthy tissues are maintained and restored upon disturbances.

We can learn about the molecular basis of animal tissue homeostasis, and its implications for health and disease, by investigating wounding responses and regeneration throughout the animal kingdom^8–11^. Some animals that can reconstruct extensive body parts, like anthozoan cnidaria (*Hydra* sp., *Nematostella vectensis*), planaria (*Schmidtea mediterranea*, *Dugesia japonica*) or zebrafish (*Danio rerio*), have been the predominant study systems for understanding the mechanisms of tissue repair^9,12–15^ and recent studies suggest that they may share common molecular features at the early points of regeneration^16,17^, at the time of the recognition and signaling of the wound^16,18,19^. However, beyond these classical model systems, we know very little about the molecular mechanisms involved in animal response to wounding.

Marine sessile animals like sponges or corals are particularly susceptible to chronic wounds caused by grazers. These wounds can yield disease^20^ and/or magnify the detrimental effects of other stressors^21^. Yet, in comparison to abiotic environmental pressures like warming or acidification, the impact of grazing is often overlooked^21–23^. As a result, our understanding on how these sessile animals respond to grazing at the molecular level is scarce. Here we focused our attention on marine sponges (Phylum Porifera) because they play a key role in the ecosystem^24–26^, they belong to the group of animals with high regenerative capacity^27^, and constitute one of the earliest-diverging animal lineages^28,29^; but, compared to other animal groups, they have been little studied^30^. Sponge wounding response and healing research mainly focused on the cellular processes involved in reparation of small incisions. This response likely varies among sponge species, but typically lasts several days and engages different cell populations migrating to the wounded area and undergoing differentiation^31–34^. At molecular level, studies focused on specific genes^35^ or lacked enough replicates for differential gene expression analysis at transcriptome level^36^. Thus, molecular processes regulating wounding responses in sponges remain to be characterized.

**Figure 1:**
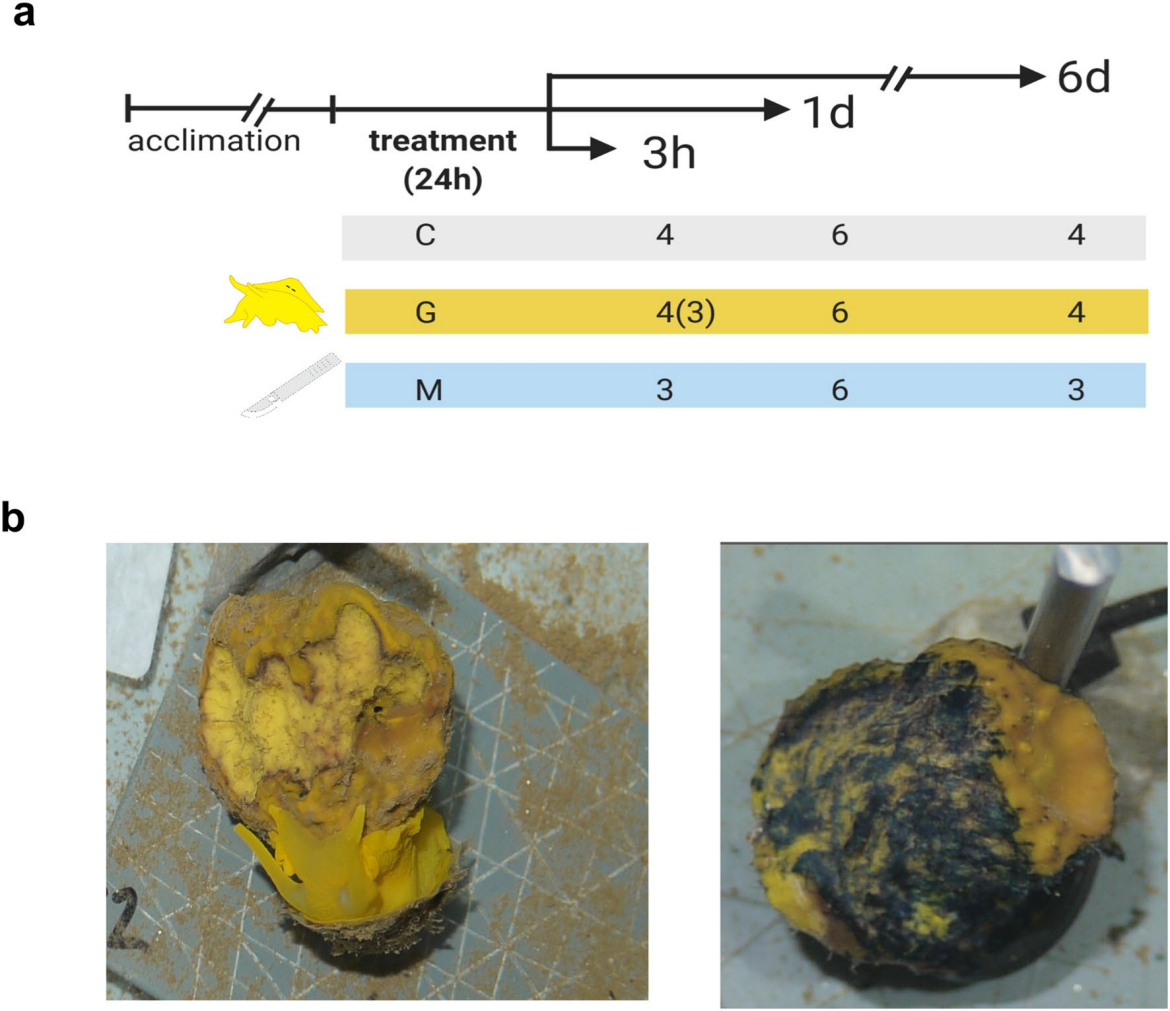
Experimental design. (**a**) Sponge individuals were divided into three specimens and let acclimate for 1 week. The specimens of each sponge individual were randomly assigned to either control (C), grazing (G), or mechanical damage (M) treatment^40^. Each experiment corresponds to a different time point after stop of treatment: 3 h (3 h); 1 day (1 d) and 6 days (6 d). Number of replicates are shown. One grazed sample collected at 3 h presented low-quality reads and was not included in the differential gene expression analysis. Created with Biorender.com. (**b**) Example of grazed (left) and mechanically-damaged (right) sponges.

Here, we investigated the injury-induced transcriptomic response of the sponge *Aplysina aerophoba* (Nardo, 1833). We applied two different wounding treatments: grazing caused by its primary predator, the opisthobranch *Tylodina perversa* (Gmelin, 1791), (i.e., grazing treatment), and repeated clipping of the sponge surface with a scalpel (i.e., mechanical damage treatment). The grazing treatment offers an ecological-relevant context of wound repair^37–40^, while mechanical damage allows us to control wound size and periodicity. In our previous study^40^, we ran three experiments to collect samples at 3 h, 1 d, and 6 d post-treatment, respectively (Fig. 1). We found a great resemblance in the chemical and cellular response of *A. aerophoba* to wounding caused by grazing and by mechanical damage, which we defined as a “jack-of-all-trades” defense strategy^40^. Now we performed differential gene expression analysis on RNA-seq data on those samples to answer the following research questions: (i) does grazing induce a similar transcriptomic response as mechanical damage, confirming the unspecific response to the specialist spongivorous? and (ii) does the wound-induced transcriptomic response in the sponge reveal conserved molecular features of animal tissue homeostasis?

## Materials and methods

### Specimen collection and experimental set-up

The samples for this study come from the experiments described in Wu et al.^40^. In short, the sponge *Aplysina aerophoba* and the opisthobranch *Tylodina perversa* were collected by Scuba diving in the Mediterranean coast of Spain (42.29408 N, 3.28944 E in 2016 and 42.1145863 N, 3.168486 E in 2017), at a depth between 2 and 10 m. Animals were transported to the Experimental Aquaria Zone at the Institute of Marine Science (ICM-CSIC) in Barcelona (Spain). There, each sponge individual was divided into 2–3 specimens with its own osculum, by applying a clean cut. Each specimen was placed into individual aquaria (6 L) maintained in a flow-through system with direct intake of seawater. After 1 week acclimation, the specimens from each sponge individual were randomly assigned to one of the following three treatments (Fig. 1), (i) control: no treatment, (ii) grazing: one sea slug, which starved for 24 h, was placed in direct contact to the sponge specimen and allowed to feed freely for 24 h, and (iii) mechanical damage: the sponge specimen was repeatedly clipped with a scalpel for 3 min every half hour at the first 3 h and the last 3 h (in the process, the ectoderm at the wound was removed). All treatments were stopped after 24 h. This design was applied in independent experiments that differ in the sampling points after stop of treatments (and use different sponge individuals). In the first experiment, samples were collected 3 h after stop of the treatment; in the second experiment after 1 d; and in the third experiment, after 6 d. The 6 d experiment was performed in 2016, the 3 h and 1 d experiments were performed in 2017.

### RNA extraction and sequencing

A total of 40 samples from 3 h, 1 d, and 6 d experiments were collected for RNA-seq (Fig. 1). Samples from grazing and mechanical damage treatments were collected right at the wound, washed with artificial seawater, and fixed in RNA*later*™ (Invitrogen, Thermo Fisher Scientific, Germany) at 4 °C overnight. Samples were then stored at − 80 °C until RNA extraction. Total RNA was extracted and processed as in^41^. In short, Total RNA was obtained by using the AllPrep DNA/RNA Mini Kit (Qiagen, Germany) and treated with DNA-free DNase Treatment and Removal Reagent (Ambion, Germany). DNA contamination was further checked by PCR amplification of eukaryotic 18S rRNA gene (Sigma-Aldrich, primers by Stewart et al*.*^42^). Total RNA was checked for purity, quantity, and quality by NanoDrop 2000c Spectrophotometer (peolab, Germany), Qubit 2.0 (Life Technologies, Carlsband, CA), and Experion™ Electrophoresis Station (Bio-Rad, Hercules, CA). Only the total RNA which was DNA-free and presented OD260/280 > 1.8 (Nanodrop) as well as RIN > 8 (Experion) was subsequently used for RNA-seq. Equal amounts of the total RNA (500 ng) were used for library construction with the TruSeq stranded mRNA library prep kit (Illumina, Inc., USA), including a poly-A enrichment step. Samples were sequenced (150 base paired-end) on the HiSeq 4000 system (Illumina, Inc., USA). Library preparation and sequencing was performed at the IKMB in Kiel, Germany.

### Differential gene expression analysis

Differential gene expression analysis was performed following the protocol described by Pita et al.^41^. Raw Illumina RNA-seq reads were qualitatively trimmed and filtered to remove adapters and low-qualitative reads in Trimmomatic-version 0.38^43^, parameters: TruSeq3-PE-2.fa:2:20:10 LEADING:3 TRAILING:3 SLIDINGWINDOW:4:15 MINLEN:36. Prokaryotic and microbial eukaryotic reads were filtered in the classifier Kaiju-version 1.6.2^44^, in greedy-5 mode (database accessed in February 2018). Because of the lack of a reference genome for *A. aerophoba*, the reads from all samples were then combined to create a de novo reference transcriptomic assembly in Trinity-version 2.6.6^45^. Assembly statistics were obtained in Trinity and TransRate-version 1.0.3^46^. Completeness of the de novo reference transcriptome was assessed by comparing the assembly against the Metazoa reference data in BUSCO-version 3^47^, trans- mode. Functional annotation was performed following Trinotate-version 3.0.2 pipeline^48^ (e-values < 1 e^−5^): first, open reading frames (ORFs) were identified in the transcript sequence (Transdecoder) and those ORFs codifying for proteins > 100 amino acids were further characterized in homology searches to publicly available data (BLAST + /SwissProt), protein domain identification (HMMER/Pfam), protein signal peptide and transmembrane domain prediction (signalP/tmHMM), as well as eggNOG, Gene Ontology (GO), and KEGG annotation^49^. Those contigs with blastx or blastp matches to Bacteria, Archaea, or Virus were further removed from the reference assembly.

Gene quantification was estimated based on Trinity component abundances by RSEM bowtie2-based quantification (version 1.2.19). Differential gene expression analysis within each experiment (i.e., 3 h, 1 d, and 6 d) was performed in edgeR as implemented in Trinity-version 2.6.5 (default parameters). Differentially expressed genes (DEGs) in grazing versus control and mechanical damage *vs* control were defined by False Discovery Rate –corrected (FDR) *p* value < 0.005 and log_2_|fold change|≥ 2 expression (fourfold change).

Based on the set of DEG for each experiment, a Gene Ontology (GO) enrichment analysis was performed in blast2GO-v5^50^. GO-annotated genes in the reference transcriptome were used as reference set. Enrichment was determined by Fisher’s exact test (FDR *p* value < 0.005). Enriched GO terms were then analyzed in REVIGO^51^ for reducing redundancy and grouping them based on semantic similarity (semantic similarity measure: SimRel). The results from differential gene expression analysis were visualized with the package ggplot2^52^ in R-version 3.5.1 (R Core Team, 2019) as implemented in R-Studio^54^. Final layout of the figures was done in Inkscape http://inkscape.org.

### Protein interaction networks in STRING

The translated coding regions of 3 h post-mechanical damage DEGs (Transdecoder; > 100 amino acids) were compared to the proteome of *A. queenslandica* (Uniprot UP000007879_444682) by Blastp (e-value < 1e^−5^). The best blastp match in *A. queenslandica* was used as input in STRING v11^55^ for protein–protein network analysis (medium required interaction score = 0.400). In addition, we highlighted those reciprocal Blastp best hit (rBBH; e-value < 1e^−5^), identified as in^56^. rBBH analysis (protocol as in Pritchard et al., 2018) of 3 h post-mechanical damage DEGs was also performed against the human reference proteome (UP000005640_9606). Those human proteins with reciprocal Blastp best hit (e-value < 1e^−5^) were then used as input for protein–protein network analysis in STRING v11^55^. The genes codifying for those proteins were searched in COSMICS v91, https://cancer.sanger.ac.uk^57^) and in the set of key cancer regulators identified by Trigos et al.^58^. Final layout of the figures was done in Inkscape http://inkscape.org.

## Results

### Overview RNA-seq analysis

We used RNA-seq to characterize the molecular response of the sponge *A. aerophoba* to wounding by grazing and by mechanical damage compared to control sponges on the samples derived from^40^. Each of the three experiments corresponds to a different sampling time point (Fig. 1): 3 h, when we expected to detect most of the signaling response based on other transcriptomic studies^41^; 1 d, concomitant to our observation of cell accumulation at the wound^40^; and 6 d, when signs of tissue recovery were obvious in all wounded sponges, although scars were still evident. Some sponges showed signs of recovery 3 d post-treatment (Supplementary Fig. S1 online)^40^.

We sequenced a total of 40 samples of *A. aerophoba*, corresponding to between 3 and 6 biological replicates per treatment (Fig. 1). The number of paired-end Illumina reads generated in this study is summarized in Supplementary Table S1 online. The de novo reference transcriptomic assembly comprised 459,466 genes (Trinity components^45^), representing 92.2% of the 902 core Metazoan genes, and 60.6% of these were complete (BUSCO^47^). The statistics of the resulting reference assembly are summarized in Supplementary Table S2 online. Overall, 72.32 ± 3.19% (average ± standard error) of the reads of each sample aligned to the de novo-assembled reference transcriptome. One grazed sample collected after 3 h (H15407-L1_S56) had a lower alignment rate of 53.53% as well as a lower number of read pairs than the other samples. Therefore, we discarded this sample for downstream analysis. The results from the differential expression analysis in edgeR as well as the annotation of the DEGs are reported in Supplementary Data S1 online (3 h experiment) and Supplementary Data S2 online (1 d experiment).

### The response of the sponge upon grazing was similar to the response upon mechanical damage

**Figure 2:**
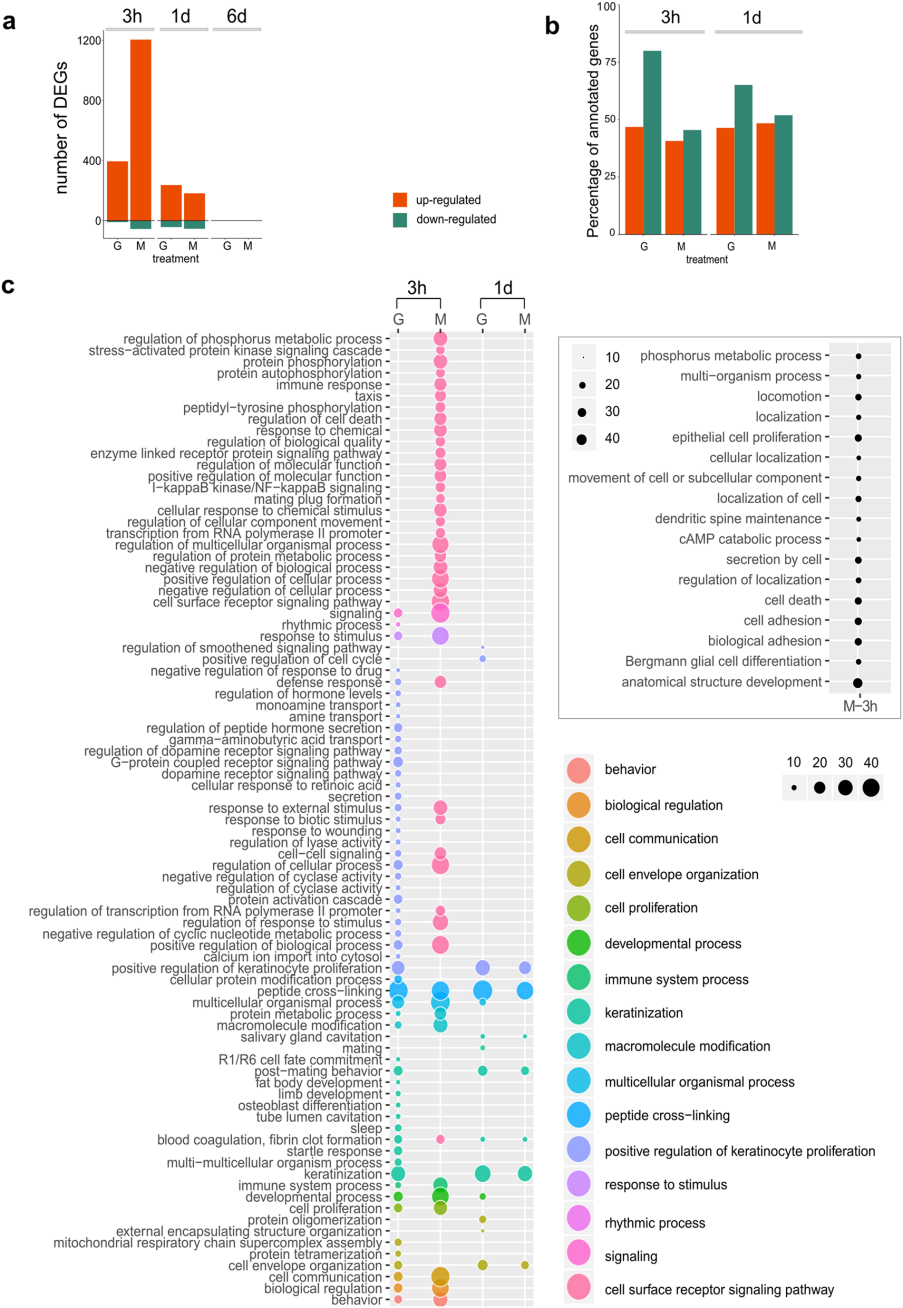
Activation of gene expression upon wounding. (**a**) Number of up- and down-regulated DEGs in grazing (G) and mechanical damage (M) groups compared to control group at 3 h, 1 d, and 6 d post-treatment experiments. Note that none DEG was detected at 6 d. “Up-regulated” refers to those genes with higher expression levels in the corresponding treatment than in control; “down-regulated” refers to those genes with lower expression levels in treatment than in control. DEGs were defined according to FDR *p* value < 0.005 and log_2_ |FC|≥ 2 expression, as calculated in edgeR. (**b**) Percentage of DEGs with annotation per time point and comparison. Annotation was performed following Trinotate pipeline. (**c**) GO categories enriched in response to each treatment at each time point. The enrichment of a GO term is indicated by a circle. Circle color corresponds to the semantic cluster as defined in REVIGO^51^ and circle size reflects Fisher test’s absolute log_10_(FDR-corrected *p* value); that is, a value of 10 means a FDR-corrected *p* value of 1e^−10^. In this sense, this bubble chart is equivalent to the tree maps generated by REVIGO^51^. Separate panel refers additional GO terms enriched in mechanical damage after 3 h. Significance threshold for enrichment test: FDR *p* value < 0.005. REVIGO dispensability threshold < 0.5.

Each wounding treatment was compared to control sponges, within each time point. We found strong activation of gene expression, both in the 3 h as well as the 1 d post-treatment experiment (Fig. 2). However, 6 d post-treatment, gene expression levels in grazing and mechanical damage samples resembled those in control (all FDR *p*-values > 0.1) (Fig. 2). At 3 h post-treatment, mechanical damage seemed to activate a stronger gene regulation than grazing, in terms of number of genes and expression levels (Fig. 2; Supplementary Figs. S2 and S3 online). However, most of the genes detected as unique to mechanical damage also showed elevated expression in grazing (Supplementary Figs. S2 and S3 online). Moreover, the functional categories (GO terms) enriched upon mechanical damage and grazing were similar, although genes related to signaling were more prominent 3 h post-mechanical damage treatment (Fig. 2c). For example, in the mechanical-damage response additional transcription factors were activated, including the transcription factors *Runt* and *Sox* as well as a *NFκß-like* gene (Supplementary Data S1 online).

From now on, we present the transcriptomic responses according to the set of annotated DEGs, which comprised ca. 46% of the total of DEGs (FDR *p* value < 0.005) (Fig. 2b).

### Wound-induced signaling pathways

**Figure 3:**
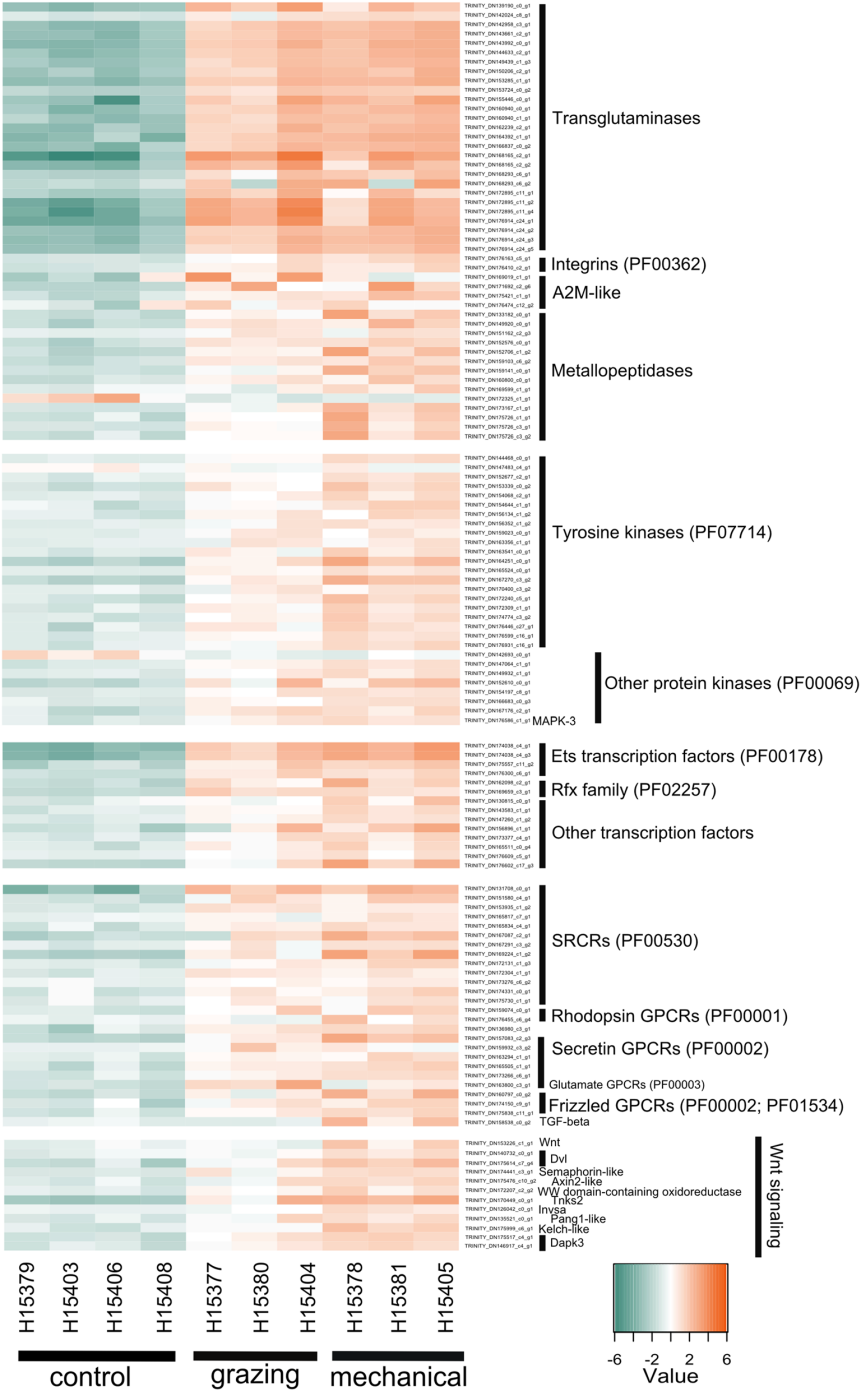
Expression changes of differentially expressed genes involved in clotting-like cascades and signaling during sponge wounding response. Heatmaps showing log_2_-transformed median-centered TMM-normalized expression changes of selected DEG (rows) in each sample from 3 h post-treatment experiment. Differential gene expression analyses were performed in edgeR following Trinity pipeline^45^. Genes were annotated in Trinotate^48^. The domain annotation (PFAM) is shown in brackets. The full list of DEGs is found in Supplementary Data S1 online.

**Figure 4:**
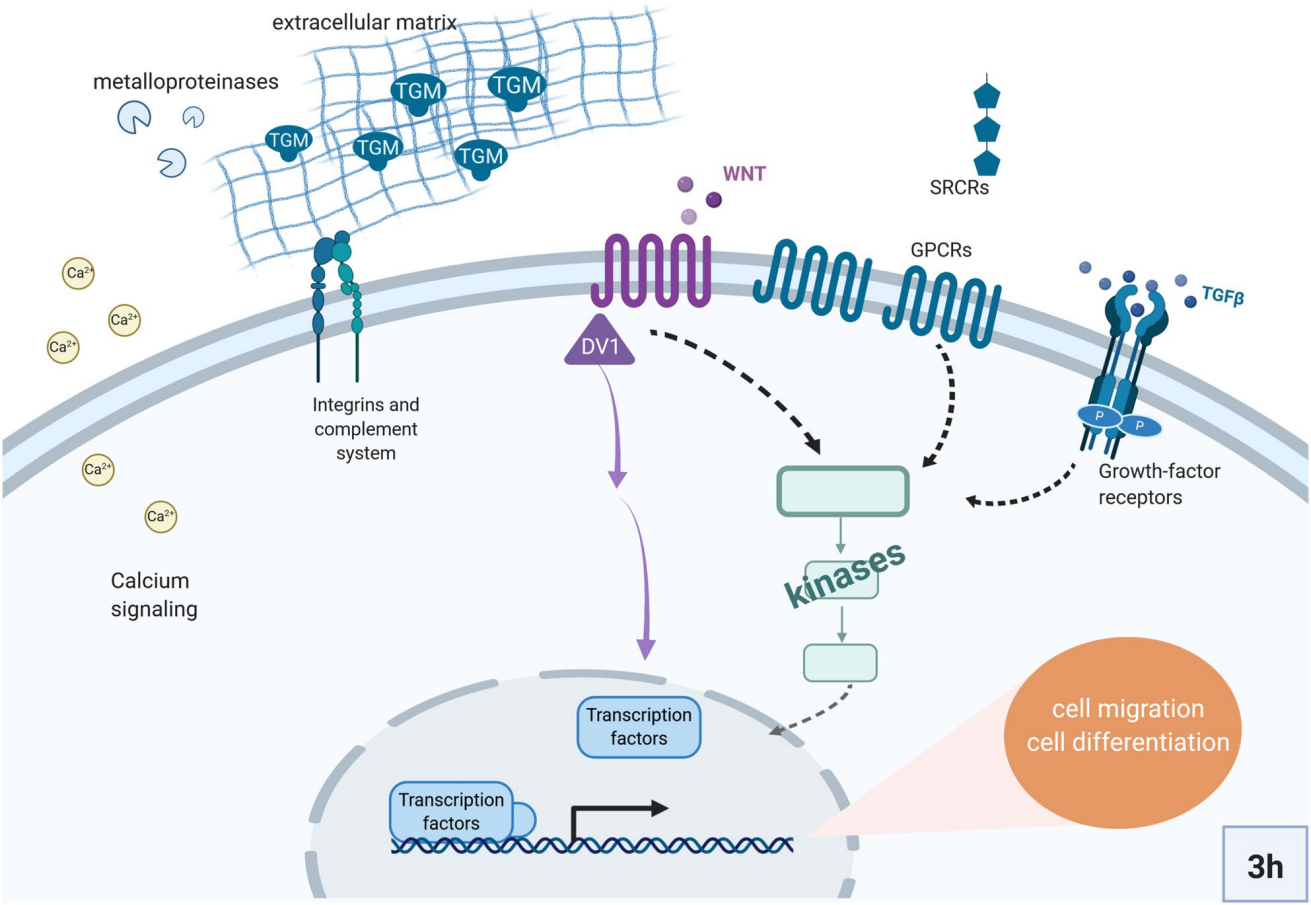
Suggested reconstruction of the sponge response to wounding at 3 h. Schematic representation based on the set of annotated DEGs. The localization of the proteins and their interactions are proposed according to literature or annotation of transmembrane domains. We hypothesize that the observed transcriptomic response may regulate cell migration and differentiation. TGM: transglutaminases. Created with BioRender.com.

Grazing and mechanical damage treatments induced a clotting-like cascade^59–61^. One of the most enriched functions both at 3 h and 1 d post-treatment experiments was the molecular function “protein-glutamine gamma-glutamyltransferase activity”, related to the GO terms “keratinization” and “peptide cross-linking” (Fig. 2c). This function corresponds to the activation of genes annotated as transglutaminases (Fig. 3). The transcriptomic response retrieved both in 3 h and 1 d experiments also included many genes involved in calcium signaling, in extracellular matrix rearrangement, and cell adhesion (e.g., integrins, cadherins, fibrillin), (Supplementary Data S1 online). At 3 h we additionally detected the activation of multiple metalloproteases, which are usually involved in degradation/deposition dynamics of the extracellular matrix, and multiple protein kinases and transcription factors (mainly Ets (PF00178) and Rfx (PF02257)-containing genes) (Fig. 3). Wounding also induced the regulation of receptors, in particular Scavenger receptors cysteine rich (SRCRs) and G-protein coupled receptors (GPCRs) (Fig. 3). A total of 13 and 8 *SRCR* genes were identified within the 3 h and 1 d DEG dataset, respectively, based on the presence of one or multiple SRCR domains (PF00530) (Fig. 3). GPCRs were identified and classified according to the 7 transmembrane domain (7tm) and included several members of the Adhesion (7tm_ 2, PF00002) and Rhodopsin (7tm1_PF00001) GPCR families, as well as one gamma-aminobutyric acid type B receptor (7tm_3, PF00003) (Fig. 3). We have summarized the set of genes that were activated in response to grazing and to mechanical-damage in 3 h experiment in a schematic representation (Fig. 4), which, we propose, summarizes the key components of the sponge response to wounding.

The response to both grazing and mechanical damage detected in 1 d experiment resembled the one reported in 3 h experiment, particularly in terms of transglutaminase-like genes and proteases (GO terms “keratinization” and “peptide cross-linking”, Fig. 2c). However, after 1 d, most of the genes related with signaling cascades (e.g., kinases, proteases) are not significantly regulated (Fig. 2c; Supplementary Data S1 online; Supplementary Fig. S4 online).

### STRING protein–protein interaction networks

**Figure 5:**
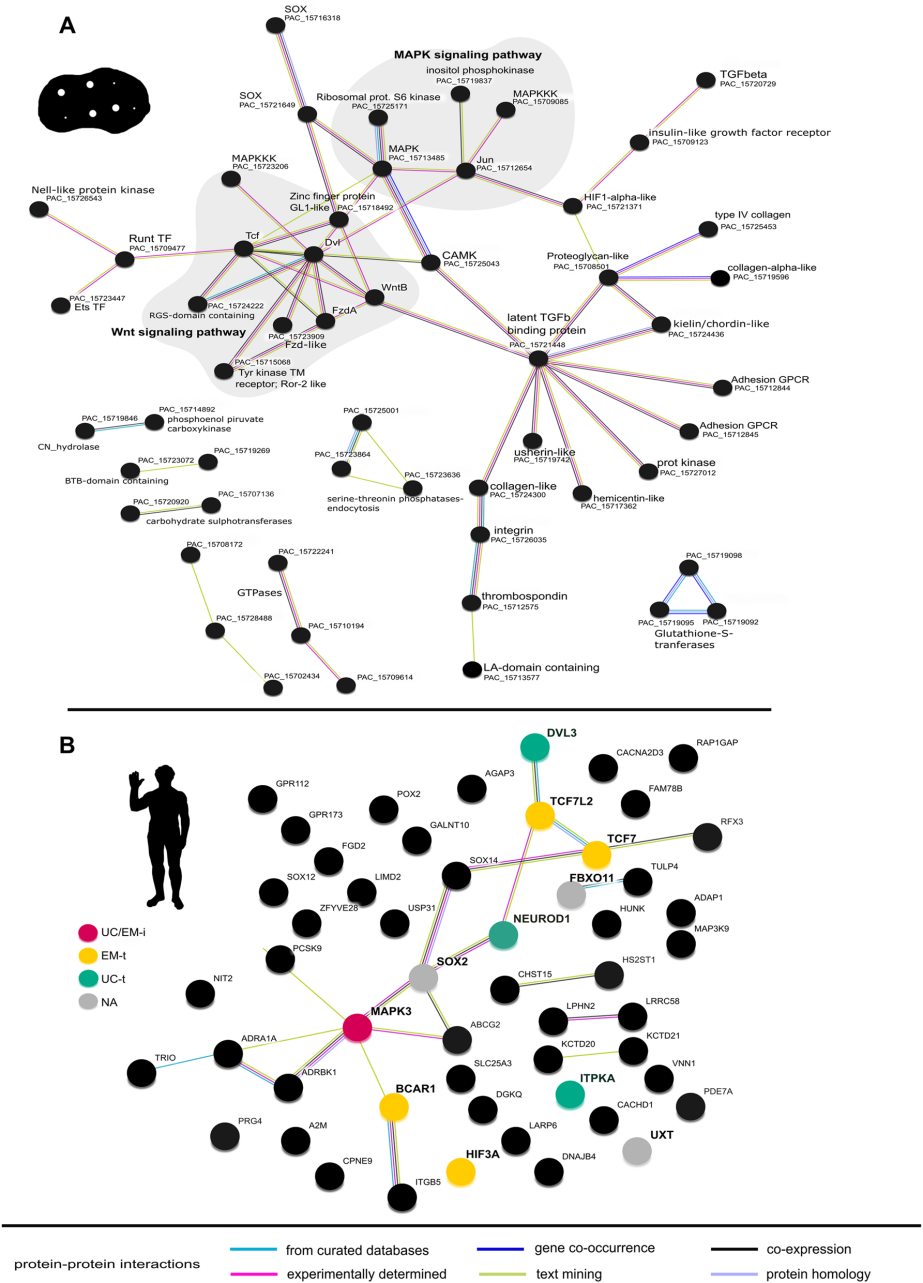
Sponge response to mechanical damage, 3 h post-treatment, involves highly conserved genes. Protein–protein network analysis on STRING v11^55^ (interaction score: 0.400). (**A**) Network of *A. queenslandica* (UP000007879_400682) proteins that best matched the 3 h mechanical-damage DEGs (blastp: e-value < 1 e^−5^). Only connected nodes are depicted. Node labels report the STRING ID of the protein and, when available, the corresponding annotation in *A. aerophoba* (e.g., CAMK PAC_15725043). Those genes identified as reciprocal blastp best hits are shown in bold. (**B)** Network of human proteins identified as orthologous of the 3 h mechanical-damage DEGs by rBHB (blastp: e-value < 1e^−5^; reference *Homo sapiens* UP000005640_9606). All DEGs with human orthologous belonged to the up-regulated set. Cancer-related genes (COSMIC v91 https://cancer.sanger.ac.uk^57^) are shown in bold. Key network regulators as in Trigos et al.^58^ are color-coded according to the phylostrata assignment in^58^. UC-t: regulator of unicellular gene target; EM-t: regulator of early metazoan gene target; UC/EM-i: regulator at the interface of unicellular/early metazoan gene networks or affecting both unicellular and early metazoan gene targets. NA: phylostrata assignment not available. Animal silhouettes from Phylopic. Legend provides the source of evidence for the interaction. Blastp hits and STRING annotations are provided in Supplementary Data S3 online.

We created a putative protein–protein interaction network of the DEGs in 3 h post-mechanical damage treatment in STRING^55^, based on the blastp protein hits to the proteome of *A. queenslandica*, the sponge species of reference in public genomic databases (Fig. 5A). STRING analysis revealed three major signaling cascades in the response to wounding: MAPK signaling pathway, Wnt signaling pathway, and the clotting-like cascade mediated by thrombospondin and other components of the extracellular matrix (Fig. 5A). A member of the Wnt signaling pathway, DVL (disheveled), could potentially mediate the crosstalk with the MAPK pathways via MAPK3 (Fig. 5A). These pathways are also connected to other receptors via a calcium/calmodulin-dependent protein kinase (CAMK, Fig. 5A). Another prominent hub was a CUB and Sushi domain-containing (CSMD3-like) protein, which appeared connected to transmembrane receptors and multiple proteins of the extracellular matrix (latent TGFβ binding protein, Fig. 5A; Supplementary Data S1 online).

Gene function evidence is scarce for sponges; thus, the network generated in STRING relies on information for similar genes/putative orthologs in other organisms. This means that they likely represent conserved gene–gene and protein–protein interactions in animal evolution. In fact, we identified 53 putative human orthologous of the 3 h post-mechanical-damage DEGs by reciprocal blastp search (rBBH, e-value < 1 e^−5^); including several GPCRs, GTPases, kinases, and multiple transcription factors (Fig. 5B). 11 out of these 53 human proteins are codified by genes listed in the Catalogue of somatic mutations in cancer (COSMIC v.91 https://cancer.sanger.ac.uk^57^) (Fig. 5B, in bold). Within these, we found eight genes defined as key regulators of ancient gene networks by Trigos et al. ^58^; three of them appeared as hubs in the *A. queenslandica*-based network (i.e., Dvl, Tcf, and MAPK3) (Fig. 5).

## Discussion

As one the earliest diverging animal phyla still extant^28,29^, sponges offer unique information on the evolution of the molecular toolkit for tissue homeostasis; yet, they remain among the least-studied animals^30^. In addition, sponges are sessile animals exposed to encounters with grazers or competitors that may cause chronic wounds (e.g.,^20,23,38^). Our results showed that the transcriptomic response to grazing is similar to the response to mechanical damage, supporting our hypothesis of an unspecific defense response against the specialist spongivorous^40^. Wounding activated the expression of SRCRs and GPCR receptors and a clotting-like cascade mediated by transglutaminases and metalloproteinases. Overall, the transcriptomic response of the sponge to wounding resembles that activated during early steps of animal whole-body regeneration^17^. Within the set of sponge genes regulated upon wounding, we also detected putative orthologs of cancer-related human genes. Previous studies predicted that those human genes have an ancient origin, according to phylostratographic and gene network analysis^58,62,63^. Our results emphasize the potential of sponge wound healing research to provide new perspectives into the function and interactions of conserved animal genes involved in fundamental cell and tissue processes.

The molecular response of the sponge upon wounding by the specialist grazer *T. perversa* greatly overlapped with the response upon mechanical damage, which agrees with the similar cellular and chemical responses we reported previously^40^. Despite the differences in the wound size (i.e., bigger in grazing than in mechanical-damage, Supplementary Fig. S1 online) and the periodicity of it (i.e., tightly controlled in the mechanical damage compared to grazing), both treatments induced similar transcriptomic responses in terms of activated functions (Fig. 3, Supplementary Fig. S3 online). This was also evident when we directly compared grazed *vs* mechanical-treated sponges (Supplementary Table S3; Supplementary Fig. S5 online). Thus, these results suggest that the sponge has not evolved a specific tolerance to its specialist grazer. Information of grazing pressure in the *Tylodina-Aplysina* interaction is limited to a single location^39^. There, > 50% sponges in the shallow waters (first 10 m, the preferred habitat of the sponge) hosted 1, occasionally 2 opisthobranchs^39^. It could be that this predation pressure is not lethal per se, but may cause chronic wounds with detrimental effects on sponge health. For example, Caribbean *Aplysina fistularis* looked diseased as a consequence of sustained grazing by cowries^20^. Our study shows that wounding cues strongly induced gene expression at the wound site (Figs. 2, 3), which could constitute a chronic response and significant metabolic burnt in the field^38^, particularly if grazing pressure is high. Overall, we need more studies to evaluate the impact of grazing on sponges as an additional stressor that can compromise sponge health, particularly in times of anthropogenic-driven environmental changes.

In our experiments, sponges were divided into clones 1 week before the experiments in order to achieve replicates. This manipulation may affect sponges in a way that could mask differences in the transcriptomic pattern of control and treated sponges in the 6 d experiment. In our current setup, we cannot completely rule out this possibility. However, we find more likely that the lack of differentially-expressed genes between treated and control samples 6 days post-treatment is due to a “return-to-baseline” situation, based on two main arguments. First, the division of sponges for making the clones is very different from the wounding treatment. The clones are generated by a clean cut between sponge chimneys at the base of the sponge, whereas mechanical damage consists on repeated clipping and affects the first 3–5 mm from the surface to the inside of the sponge. Second, we are collecting the samples at the wounding site, which does not overlap with the place sponges were cut for generating the clones. Nevertheless, we are currently working in characterizing the response of the sponge in the latter time points (6 days post-treatment and beyond) to get a better understanding of the recovery phase.

We defined sponge molecular response to wounding as a “clotting-like” cascade^59–61^ because we found multiple DEGs annotated as transglutaminases both in grazing and mechanical-damage treatments and in the 3 h as well as the 1 d experiment, resembling the molecular cascades involved in clot formation and stabilization in other animals, including vertebrates^60,61^ and invertebrates^59^. We propose a reconstruction of the main genes involved in sponge response to wounding (Fig. 4) and hypothesize that this gene regulation is mediating cellular migration based on reports in other animals^64,65^ and on the observation of spherulous cell accumulation at the wound in *Aplysina* sponges, particularly 1 d post-treatment^33,40^. In addition, the observed activation of Ets-domain transcription factors may point to differentiation by increased “pinacocyte-like” expression signature at the wound. Single-cell RNA-seq from the sponge *A. queenslandica* revealed that Ets-domain transcription factors are enriched in pinacocytes, sponge epidermal cells^66^. The family of Ets-domain transcription factors is considered a metazoan novelty^67^, with conserved roles in cellular differentiation and development in animals^68,69^. It is difficult to unequivocally distinguish different cellular processes (i.e., migration, proliferation, and differentiation) based on gene expression patterns alone. Yet, migration and differentiation prevail at the wound in different sponge species^31–33^, whereas proliferation seems more relevant in adjacent areas^34^. Further studies are necessary to connect the molecular responses to cellular processes in sponges.

Wounding-induced gene expression patterns in the sponge resembled the early steps of regeneration in sponges and other animal phyla. The signaling pathways involved in the 3 h post-treatment transcriptomic response are similar to those identified at the onset of whole-body regeneration in sea starts (3 h post-treatment), *Hydra* (3 h post-treatment), and planaria (12 h post-treatment), which typically correspond to the wound response phase^14,17,70^. In particular, they overlap in the regulation of MAPK and calcium signaling, and transcription factors^17^. Another common player is the Wnt signaling pathway, which determines patterning in whole-body regeneration as well as in animal development (e.g.,^17,71^ and references therein). In sponges, Wnt pathway is involved in both development^72^ and regeneration^27^. Recently, Soubigou et al.^27^ monitored the morphological and gene expression changes involved in the regeneration of adult disassociated cells into a new functional sponge during the first 24 d after disassociation. They found strong correlation of the pathways involved in sponge regeneration and in sponge post-larval development^27^. Despite the differences in the degree of damage and sampling time points, several of the genes and pathways in sponge regeneration identified by Soubigou et al*.*^27^ are also induced by wounding in our study, like the Wnt pathway, the TGFβ signaling, and genes involved in cell adhesion and extracellular matrix rearrangement. Overall, the transcriptomic response of the sponge to wounding agrees with conserved genetic features involved in tissue repair in animals with high regeneration capacity^14,16,17,27,70,73,74^.

Sponge response to wounding revealed key genes in maintaining animal tissue homeostasis, from sponges to humans. In the set of proteins codified by sponge genes responding to mechanical damage, we identified putative orthologs of human proteins involved in G-protein coupled signaling pathway, ubiquitination, and components of the MAP kinase signaling transduction pathway (Fig. 5). In humans, these proteins mediate diverse biological processes such as cell adhesion, proliferation, or differentiation (Supplementary Data S3 online). For some of these human genes, their malfunction or dysregulation has been observed during tumors (COSMIC https://cancer.sanger.ac.uk^57^, Fig. 5). Recent studies established a link between the origin of genes and gene networks and their implication in cancer^7,58,63,75^. Those genes and gene–gene interactions that appeared at the emergence of multicellularity for coordinating cell behavior may play a prominent role in cancer development, when their function is altered^7,58,63,75^. For example, TCF7L2 was predicted to function as a key regulator in networks of genes emerged in early metazoan^58^ and is a well-known oncogene (COSMIC https://cancer.sanger.ac.uk^57^). Finding similar genes in the context of sponge response to wounding adds additional support of their conserved role in regulating homeostasis. It has been previously reported that sponge genomes harbor genes related to those involved in human cancer^76,77^. As one of the oldest animal groups till extant, sponges offer a unique opportunity for understanding the evolution of the functions and interactions of these cancer-related genes. Validating gene functions in sponges is difficult due to their limited amenability to manipulation, however, wound healing has the potential to provide an experimental context with a phenotype that can be directly linked to molecular responses involved in the fundamental process of tissue homeostasis.

## Conclusion

Here, we provide the first detailed study of the transcriptomic response to wound healing in sponges. The transcriptomic response of *A. aerophoba* to grazing by *T. perversa* resembles the response to wounding caused by mechanical damage. Both wounding cues induced a strong activation of gene expression that, we hypothesize, may have a detrimental metabolic cost for the sponge. Wounding activated the Wnt signaling, MAPK signaling and “clotting-like” cascade, which resembles the onset of whole-body regeneration in sponges^27^ and other animal phyla^17^. The sponge transcriptomic response to wounding included putative orthologs of human genes that, when altered, yield cancer^58,62,63^. Cancer development constitutes a disruption of tissue homeostasis and is largely controlled by ancient molecular pathways^58,62,63^. Sponges are a key phylum to understand the emergence of animal life and we propose that sponge wound healing can offer an innovative system to research how these ancient genes and regulatory networks contribute to tissue homeostasis and animal health.

## Supplementary Information

Supplementary Information.

Supplementary Information.

Supplementary Information.

Supplementary Information.

Supplementary Information.

## Data Availability

Raw reads, metadata, and gene quantification matrices generated during this study are available in the ArrayExpress database at EMBL-EBI archive (www.ebi.ac.uk/arrayexpress) under accession number E-MTAB-8601. De novo reference transcriptome assembly is available at the European Nucleotide Archive under project PRJEB46965. The annotation of the reference assembly is provided in Supplementary Data S4 online. Further generated/processed data are included in the Supplementary Material online.

## References

[1] Eming, S. A., Martin, P. & Tomic-Canic, M. Wound repair and regeneration: Mechanisms, signaling, and translation. *Sci. Transl. Med.* **6**, 265sr6–265sr6 (2014). 10.1126/scitranslmed.3009337 PMC4973620 25473038

[2] Wilkinson HN, Hardman MJ (2020). Wound healing: Cellular mechanisms and pathological outcomes. Open Biol..

[3] Dvorak HF (1986). Tumors: Wounds that do not heal. Similarities between tumor stroma generation and wound healing. N. Engl. J. Med..

[4] Dvorak HF (2015). Tumors: Wounds that do not heal–Redux. Cancer Immunol. Res..

[5] Schäfer M, Werner S (2008). Cancer as an overhealing wound: An old hypothesis revisited. Nat. Rev. Mol. Cell Biol..

[6] MacCarthy-Morrogh, L. & Martin, P. The hallmarks of cancer are also the hallmarks of wound healing. *Sci. Signal.* **13**, eaay8690 (2020). 10.1126/scisignal.aay8690 32900881

[7] Trigos AS, Pearson RB, Papenfuss AT, Goode DL (2018). How the evolution of multicellularity set the stage for cancer. Br. J. Cancer.

[8] Bely AE, Nyberg KG (2010). Evolution of animal regeneration: Re-emergence of a field. Trends Ecol. Evol..

[9] Bosch TCG (2007). Why polyps regenerate and we don’t: Towards a cellular and molecular framework for *Hydra* regeneration. Dev. Biol..

[10] Gurtner GC, Werner S, Barrandon Y, Longaker MT (2008). Wound repair and regeneration. Nature.

[11] Slack JM (2017). Animal regeneration: Ancestral character or evolutionary novelty?. EMBO Rep..

[12] Wenger Y, Buzgariu W, Reiter S, Galliot B (2014). Injury-induced immune responses in *Hydra*. Semin. Immunol..

[13] Poss, K. D., Wilson, L. G. & Keating, M. T. Heart regeneration in zebrafish. *Science (80-. ).* **298**, 2188–2190 (2002). 10.1126/science.1077857 12481136

[14] Kao D, Felix D, Aboobaker A (2013). The planarian regeneration transcriptome reveals a shared but temporally shifted regulatory program between opposing head and tail scenarios. BMC Genomics.

[15] Gehrke, A. R. *et al.* Acoel genome reveals the regulatory landscape of whole-body regeneration. *Science (80-. ).* **363** (2019). 10.1126/science.aau6173 30872491

[16] DuBuc TQ, Traylor-Knowles N, Martindale MQ (2014). Initiating a regenerative response; cellular and molecular features of wound healing in the cnidarian *Nematostella vectensis*. BMC Biol..

[17] Cary GA, Wolff A, Zueva O, Pattinato J, Hinman VF (2019). Analysis of sea star larval regeneration reveals conserved processes of whole-body regeneration across the metazoa. BMC Biol..

[18] Owlarn S (2017). Generic wound signals initiate regeneration in missing-tissue contexts. Nat. Commun..

[19] Ramon-Mateu J, Ellison ST, Angelini TE, Martindale MQ (2019). Regeneration in the ctenophore *Mnemiopsis leidyi* occurs in the absence of a blastema, requires cell division, and is temporally separable from wound healing. BMC Biol..

[20] Pawlik JR, Deignan LK (2015). Cowries graze Verongid sponges on Caribbean reefs. Coral Reefs.

[21] Rice MM, Ezzat L, Burkepile DE (2019). Corallivory in the anthropocene: Interactive effects of anthropogenic stressors and corallivory on coral reefs. Front. Mar. Sci..

[22] Pawlik, J. R., Loh, T.-L., McMurray, S. E. & Finelli, C. M. Sponge communities on Caribbean coral reefs are structured by factors that are top-down, not bottom-up. *PLoS One* **8**, e62573 (2013). 10.1371/journal.pone.0062573 PMC3648561 23667492

[23] Mortimer C, Dunn M, Haris A, Jompa J, Bell J (2021). Estimates of sponge consumption rates on an Indo-Pacific reef. Mar. Ecol. Prog. Ser..

[24] de Goeij, J. M. *et al.* Surviving in a marine desert: the sponge loop retains resources within coral reefs. *Science (80-. ).* **342**, 108–10 (2013). 10.1126/science.1241981 24092742

[25] Rix L (2016). Differential recycling of coral and algal dissolved organic matter via the sponge loop. Funct. Ecol..

[26] Maldonado M (2015). Sponge grounds as key marine habitats: A synthetic review of types, structure, functional roles and conservation concerns. Mar. Animal Forests.

[27] Soubigou A, Ross EG, Touhami Y, Chrismas N, Modepalli V (2020). Regeneration in sponge *Sycon ciliatum* partly mimics postlarval development. Development.

[28] Telford MJ, Moroz LL, Halanych KM (2016). A sisterly dispute. Nature.

[29] Feuda R (2017). Improved modeling of compositional heterogeneity supports sponges as sister to all other animals. Curr. Biol..

[30] Dunn CW, Leys SP, Haddock SHD (2015). The hidden biology of sponges and ctenophores. Trends Ecol. Evol..

[31] Borisenko, I. E., Adamska, M., Tokina, D. B. & Ereskovsky, A. V. Transdifferentiation is a driving force of regeneration in *Halisarca dujardini* (Demospongiae, Porifera). *PeerJ* **3**, e1211 (2015). 10.7717/peerj.1211 PMC4556153 26336645

[32] Lavrov, A. I., Bolshakov, F. V., Tokina, D. B. & Ereskovsky, A. V. Sewing up the wounds: The epithelial morphogenesis as a central mechanism of calcaronean sponge regeneration. *J. Exp. Zool. Part B Mol. Dev. Evol.* **330**, 351–371 (2018). 10.1002/jez.b.22830 30421540

[33] Ereskovsky, A. V. *et al.* Transdifferentiation and mesenchymal‐to‐epithelial transition during regeneration in Demospongiae (Porifera). *J. Exp. Zool. Part B Mol. Dev. Evol.* **334**, 37–58 (2020). 10.1002/jez.b.22919 31725194

[34] Alexander, B. E. *et al.* Cell kinetics during regeneration in the sponge Halisarca caerulea: how local is the response to tissue damage? *PeerJ* **3**, e820 (2015). 10.7717/peerj.820 PMC4358696 25780772

[35] Pozzolini, M. *et al.* Insights into the evolution of metazoan regenerative mechanisms: TGF superfamily member roles in tissue regeneration of the marine sponge *Chondrosia reniformis* Nardo, 1847. *J. Exp. Biol.* **222**, jeb207894 (2019). 10.1242/jeb.207894 31371401

[36] Kenny NJ (2018). Towards the identification of ancestrally shared regenerative mechanisms across the Metazoa: A transcriptomic case study in the demosponge *Halisarca caerulea*. Mar. Genomics.

[37] Pawlik, J. R. Handbook of marine natural products. in *Handbook of Marine Natural Products* (eds. Fattorusso, E., Gerwick, W. H. & Taglialatela-Scafati, O.) 677–705 (Springer, New York, 2012). 10.1007/978-90-481-3834-0

[38] Walters KD, Pawlik JR (2005). Is there a trade-off between wound-healing and chemical defenses among Caribbean reef sponges?. Integr. Comp. Biol..

[39] Becerro MA, Turon X, Uriz MJ, Templado J (2003). Can a sponge feeder be a herbivore? *Tylodina perversa *(Gastropoda) feeding on *Aplysina aerophoba* (Demospongiae). Biol. J. Linn. Soc..

[40] Wu Y-C (2020). Opisthobranch grazing results in mobilisation of spherulous cells and re-allocation of secondary metabolites in the sponge *Aplysina aerophoba*. Sci. Rep..

[41] Pita L, Hoeppner MP, Ribes M, Hentschel U (2018). Differential expression of immune receptors in two marine sponges upon exposure to microbial-associated molecular patterns. Sci. Rep..

[42] Stewart FJ, Ottesen EA, Delong EF (2010). Development and quantitative analyses of a universal rRNA-subtraction protocol for microbial metatranscriptomics. ISME J..

[43] Bolger, A. M., Lohse, M. & Usadel, B. Trimmomatic: A flexible read trimming tool for Illumina NGS data. *Bioinformatics***btu170** (2014). 10.1093/bioinformatics/btu170 PMC4103590 24695404

[44] Menzel P, Krogh A (2015). Fast and sensitive taxonomic classification for metagenomics with Kaiju. Nat. Commun..

[45] Haas BJ (2013). De novo transcript sequence reconstruction from RNA-Seq: reference generation and analysis with Trinity. Nat. Protoc..

[46] Smith-Unna R, Boursnell C, Patro R, Hibberd JM, Kelly S (2016). TransRate: Reference free quality assessment of de-novo transcriptome assemblies. Genome Res..

[47] Simao FA, Waterhouse RM, Ioannidis P, Kriventseva EV, Zdobnov EM (2015). Genome analysis BUSCO: Assessing genome assembly and annotation completeness with single-copy orthologs. Bioinformatics.

[48] Bryant DM (2017). A tissue-mapped axolotl *de novo* transcriptome enables identification of limb regeneration factors. Cell Rep..

[49] Kanehisa M (2000). KEGG: Kyoto encyclopedia of genes and genomes. Nucleic Acids Res..

[50] Conesa A (2005). Blast2GO: A universal tool for annotation, visualization and analysis in functional genomics research. Bioinformatics.

[51] Supek, F., Bošnjak, M., Škunca, N. & Šmuc, T. REVIGO summarizes and visualizes long lists of gene ontology terms. *PLoS One* **6**, e21800 (2011). 10.1371/journal.pone.0021800 PMC3138752 21789182

[52] Wickham, H. *ggplot2: Elegant graphics for data analysis*. (Springer, Berlin, 2016).

[53] Team, R. C. R: A language and environment for statistical computing. (2019).

[54] Team, Rs. RStudio: Integrated Development for R. (2015).

[55] Szklarczyk D (2019). STRING v11: Protein–protein association networks with increased coverage, supporting functional discovery in genome-wide experimental datasets. Nucleic Acids Res..

[56] Pritchard, L., Jones, S. & Cock, P. IBioIC Introd. Bioinform. Train. Course 10.5281/zenodo.1184095 (2018).

[57] Forbes, S. A. *et al.* The catalogue of somatic mutations in cancer (COSMIC). *Curr. Protoc. Hum. Genet.* **57** (2008). 10.1002/0471142905.hg1011s57 PMC2705836 18428421

[58] Trigos AS, Pearson RB, Papenfuss AT, Goode DL (2019). Somatic mutations in early metazoan genes disrupt regulatory links between unicellular and multicellular genes in cancer. Elife.

[59] Cerenius L, Söderhäll K (2011). Coagulation in invertebrates. J. Innate Immun..

[60] Davie EW, Fujikawa K, Kisiel W (1991). The coagulation cascade: Initiation, maintenance, and regulation. Biochemistry.

[61] Richardson VR, Cordell P, Standeven KF, Carter AM (2013). Substrates of factor XIII-A: Roles in thrombosis and wound healing. Clin. Sci..

[62] Domazet-Lošo T, Tautz D (2010). Phylostratigraphic tracking of cancer genes suggests a link to the emergence of multicellularity in metazoa. BMC Biol..

[63] Trigos AS, Pearson RB, Papenfuss AT, Goode DL (2017). Altered interactions between unicellular and multicellular genes drive hallmarks of transformation in a diverse range of solid tumors. Proc. Natl. Acad. Sci. USA.

[64] Rohani MG, Parks WC (2015). Matrix remodeling by MMPs during wound repair. Matrix Biol..

[65] Grose R (2002). A crucial role of beta 1 integrins for keratinocyte migration in vitro and during cutaneous wound repair. Development.

[66] Sebé-Pedrós A (2018). Early metazoan cell type diversity and the evolution of multicellular gene regulation. Nat. Ecol. Evol..

[67] Paps J, Holland PWH (2018). Reconstruction of the ancestral metazoan genome reveals an increase in genomic novelty. Nat. Commun..

[68] Sharrocks AD (2001). The ETS-domain transcription factor family. Nat. Rev. Mol. Cell Biol..

[69] Larroux C (2006). Developmental expression of transcription factor genes in a demosponge: Insights into the origin of metazoan multicellularity. Evol. Dev..

[70] Petersen HO (2015). A comprehensive transcriptomic and proteomic analysis of *Hydra* head regeneration. Mol. Biol. Evol..

[71] Cardozo MJ, Mysiak KS, Becker T, Becker CG (2017). Reduce, reuse, recycle—Developmental signals in spinal cord regeneration. Dev. Biol..

[72] Adamska, M. *et al.* Wnt and TGF-β expression in the sponge *Amphimedon queenslandica* and the origin of metazoan embryonic patterning. *PLoS One* **2**, e1031 (2007). 10.1371/journal.pone.0001031 PMC2000352 17925879

[73] Stewart ZK (2017). Transcriptomic investigation of wound healing and regeneration in the cnidarian *Calliactis polypus*. Sci. Rep..

[74] Chablais F, Jazwinska A (2012). The regenerative capacity of the zebrafish heart is dependent on TGF signaling. Development.

[75] Chen H, Lin F, Xing K, He X (2015). The reverse evolution from multicellularity to unicellularity during carcinogenesis. Nat. Commun..

[76] Srivastava M (2010). The *Amphimedon queenslandica* genome and the evolution of animal complexity. Nature.

[77] Ćetković H, Halasz M, Herak Bosnar M (2018). Sponges: A reservoir of genes implicated in human cancer. Mar. Drugs.

