# Supplementary material for: Wounding response in Porifera (sponges) activates ancestral signaling cascades involved in animal healing, regeneration, and cancer
Source: Sci Rep. 2022 Jan 25;12:1307. doi: 10.1038/s41598-022-05230-x (PMC8789774; doi:10.1038/s41598-022-05230-x)
Supplement: Supplementary file 1 — Supplementary Information. [file 41598_2022_5230_MOESM1_ESM.docx]

Supplementary Material

Wounding response in Porifera (sponges) activates ancestral signaling cascades involved in animal healing, regeneration, and cancer

Yu-Chen Wu^1,2^, Soeren Franzenburg^3^ (0000-0001-6374-4910), Marta Ribes^4^ (0000-0001-9747-295), Lucía Pita^1^* (000-0003-0163-1587)

# Supplementary Data

**Supplementary Data 1.** DEGs in 3h experiment as obtained by edgeR (genes with FDR p-value <0.005). GeneID: Trinity component; logFC: log(fold change); Pfam: Pfam annotation; sigP: signalP annotation; blastPUniprot: Uniprot protein name for best blastp hit; blastpDescription: description of the protein name. No annotation is denoted with “.”. The count matrix for each DEG and sample is as well reported (“counts.matrix” sheet).

**Supplementary Data 2.** DEGs in 1d experiment as obtained by edgeR (genes with FDR p-value <0.005). GeneID: Trinity component; logFC: log(fold change); Pfam: Pfam annotation; sigP: signalP annotation; blastPUniprot: Uniprot protein name for best blastp hit; blastpDescription: description of the protein name. No annotation is denoted with “.”. The count matrix for each DEG and sample is as well reported (“counts.matrix” sheet).

**Supplementary Data 3.** Input data for protein-protein networks in STRING v11 (Szklarczyk et al., 2019). The tables report the DEGs at 3h post-mechanical damage with the corresponding to: i) blastp hit to *A. queenslandica* proteome (Uniprot) and STRING ID for the protein (“Aqueen”) and ii) reciprocal blastp best hit to human proteome (Uniprot) and STRING ID (“hsa”).

**Supplementary Data 4.** Full annotation of reference transcriptome assembly as generated in Trinotate pipeline (Bryant et al., 2017).

# Supplementary Tables and Figures

**Supplementary Table S1. Number of read pairs (million reads).** “Raw” refers to the output from sequencing; “Clean” to surviving read pairs after trimming and filtering in trimmomatic-v0.38; and “Eukaryote” to pairs identified as non-prokaryotic and nonmicrobial eukaryote by kaiju-v1.6.2 (Menzel and Krogh, 2015).

|  | **Raw** | **Clean** | **Eukaryote** |
| --- | --- | --- | --- |
| average per library (± standard error) | 27.0 ± 10.9 | 20.0 ± 8.5 | 13.8 ± 5.8 |
| total | 1 115.5 | 825.5 | 553.3 |

**Supplementary Table S2. Statistics of the *de novo* transcriptomic assembly from 40 samples.** Transcripts refer to Trinity isoforms, genes refer to Trinity components. Mb: mega bases.

| **Statistics:** |  |
| --- | --- |
| No. Transcripts – Trinity isoforms | 1 101 660 |
| No. Genes – Trinity components | 478 830 |
| Transcripts with open reading frames, % | 60.8 |
| Average transcript length, nucleotides | 510.8 |
| N50 | 582 |
| Total assembled bases, Mb | 562.7 |
| **Filtering after Blast filtering:** |  |
| No. Transcripts – Trinity isoforms | 1 065 137 |
| No.Genes – Trinity components | 459 466 |

**Supplementary Table S3.** **Annotated differential expressed genes in grazing in comparison to mechanical-damage.**

| **GeneID** | **logFC** | **FDR** | ***A.queen* blastp** | **Trinotate annotation** |
| --- | --- | --- | --- | --- |
| TRINITY_DN161680_c1_g1 | -4.6 | 8.21e-8 | - | TM prediction |
| TRINITY_DN158538_c0_g2 | -3.3 | 0.002 | TGFß1 (A0A1X7V271): 383 : 23.8% : 1.8e-16 | TGF-ß-like; PF00019: TGF-ß domain; signal peptide-TM |
| TRINITY_DN160114_c1_g2 | -3.8 | 0.003 |  | TM prediction |
| TRINITY_DN139241_c0_g1 | -4.4 | 0.031 | SRCR (A0A1X7U1B7): 96 : 43.8% : 1.8e-11 | TM prediction |
| TRINITY_DN151192_c3_g1 | -2.6 | 0.035 | c-Fos-like (A0A1X7TY01): 60: 45.6% : 3.0e-18 | c-Fos-like; bZIPdomain^1^ |
| TRINITY_DN151667_c2_g1 | -2.6 | 0.035 | c-Fos-like (A0A1X7TY01) : 37 : 55.3% : 1.4e-3 | ^2^ |
| TRINITY_DN148741_c1_g1 | -3.8 | 0.035 | - | TM prediction |
| TRINITY_DN173731_c4_g1 | -2.6 | 0.042 | - | c-Fos-like |
| TRINITY_DN169503_c0_g2 | 8.7 | 0.006 | Uncharacterized protein (A0A1X7VUW9): 162: 37.6%:9.0e-17 | Balbiani ring protein-like; PF02014: Reeler domain, PF03128: CXCXC repeat |
| TRINITY_DN140810_c0_g1 | 7.8 | 0.019 | IF rod domain containing protein (A0A1X7UUA0): 403:25.6%: 9.9e-17 | Filament protein-like; PF00038: Intermediate filament protein |
| TRINITY_DN169019_c1_g1 | 3.5 | 0.035 | Ficolin-like (A0A1X7U0C2): 116: 63.8%: 4.22e-48 | Tenascin/ficolin-like gene; PF00147: Fibrinogen beta and gamma chains, C-terminal globular domain |

DEGs defined as FDR p-value (FDR) < 0.05. logFC refers to log2(fold change): positive values denote activation upon grazing and negative values denote activation upon mechanical-damage. Aqueen blastp column reports the best blastp hit in *A. queenslandica* reference proteome as follows: “protein name or function (Uniprot ID): sequence overlap : percentage of identical matches : e-value” . Trinotate annotation column report results blastx/blastp protein match, PFAM annotation and sigP annotation. TM: transmembrane domain.

^1^longest ORF codifies to 98aa long protein, so no detected in Trinotate pipeline. Identified bu NCBI CDD conserved domain search, e-value: 2.2e-5

^2^longest ORF codifies to 77 amino acid- long protein, so no detected in Trinotate pipeline. Blastp in Uniprot. No conserved domain detected.

**
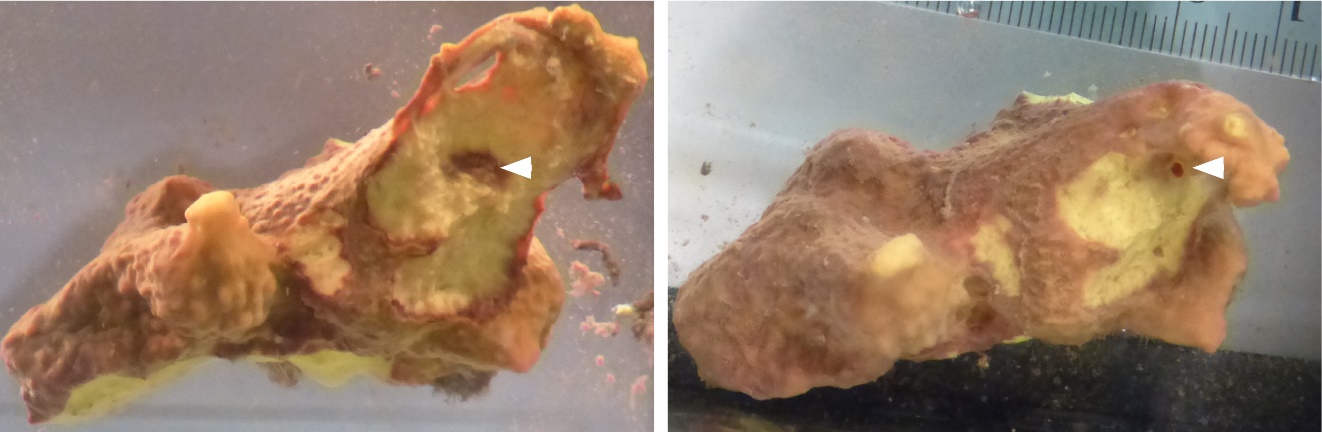
**

**Supplementary Figure S1. Wound caused by grazing.** Example of a wound caused by grazing after stop of grazing treatment (left) and scar 3d post-treatment (right). This figure was published in Wu et al. (Wu et al., 2020) as part of Supplementary Figure S3 under CC-BY license.

**
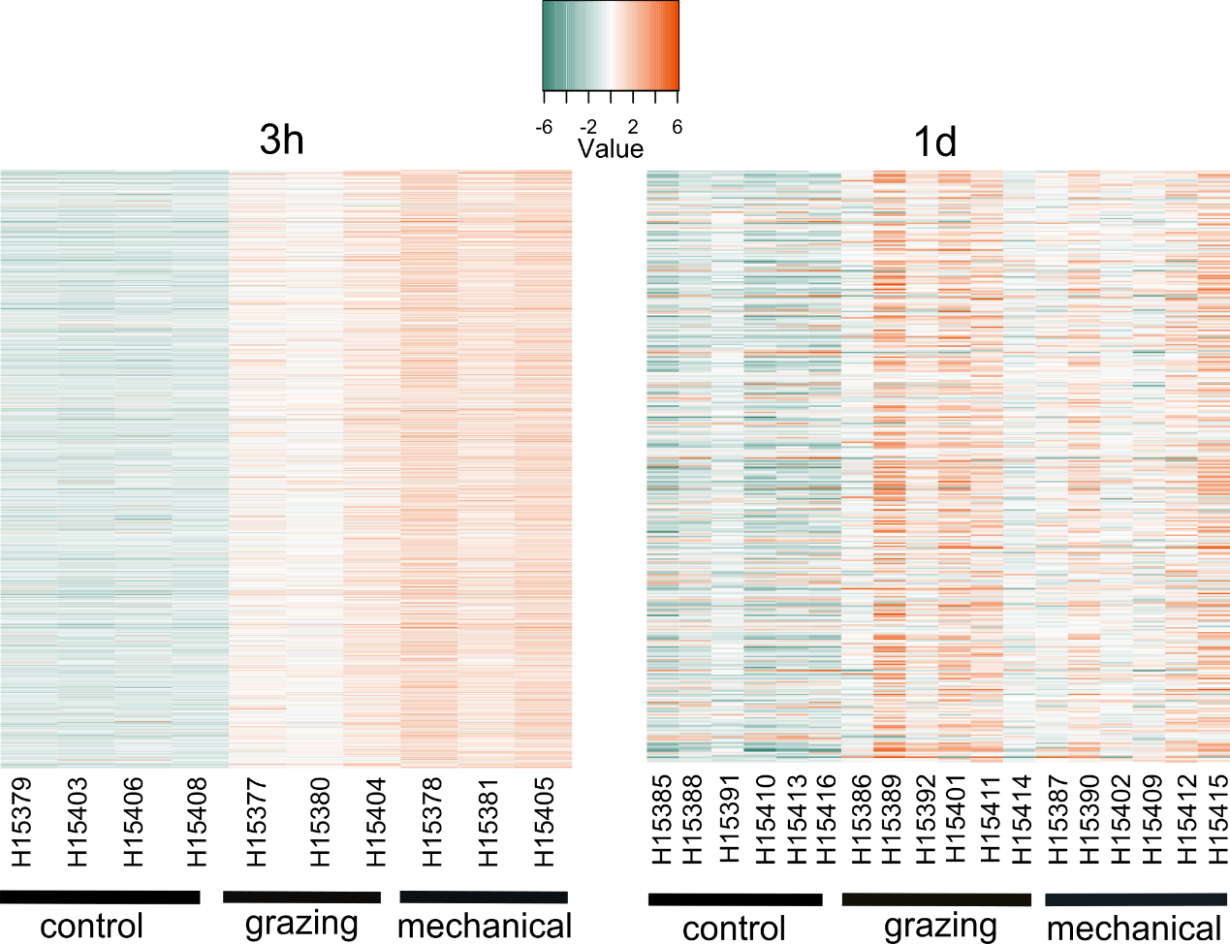
**

**Supplementary Figure S2.** **Activation of gene expression upon wounding.** Heatmaps showing log_2_-transformed median-centered TMM-normalized expression changes of each DEG (rows) in each sample from 3h (left) and 1d (right). Differential gene expression analyses were performed in edgeR following Trinity pipeline (Haas et al., 2013).

**
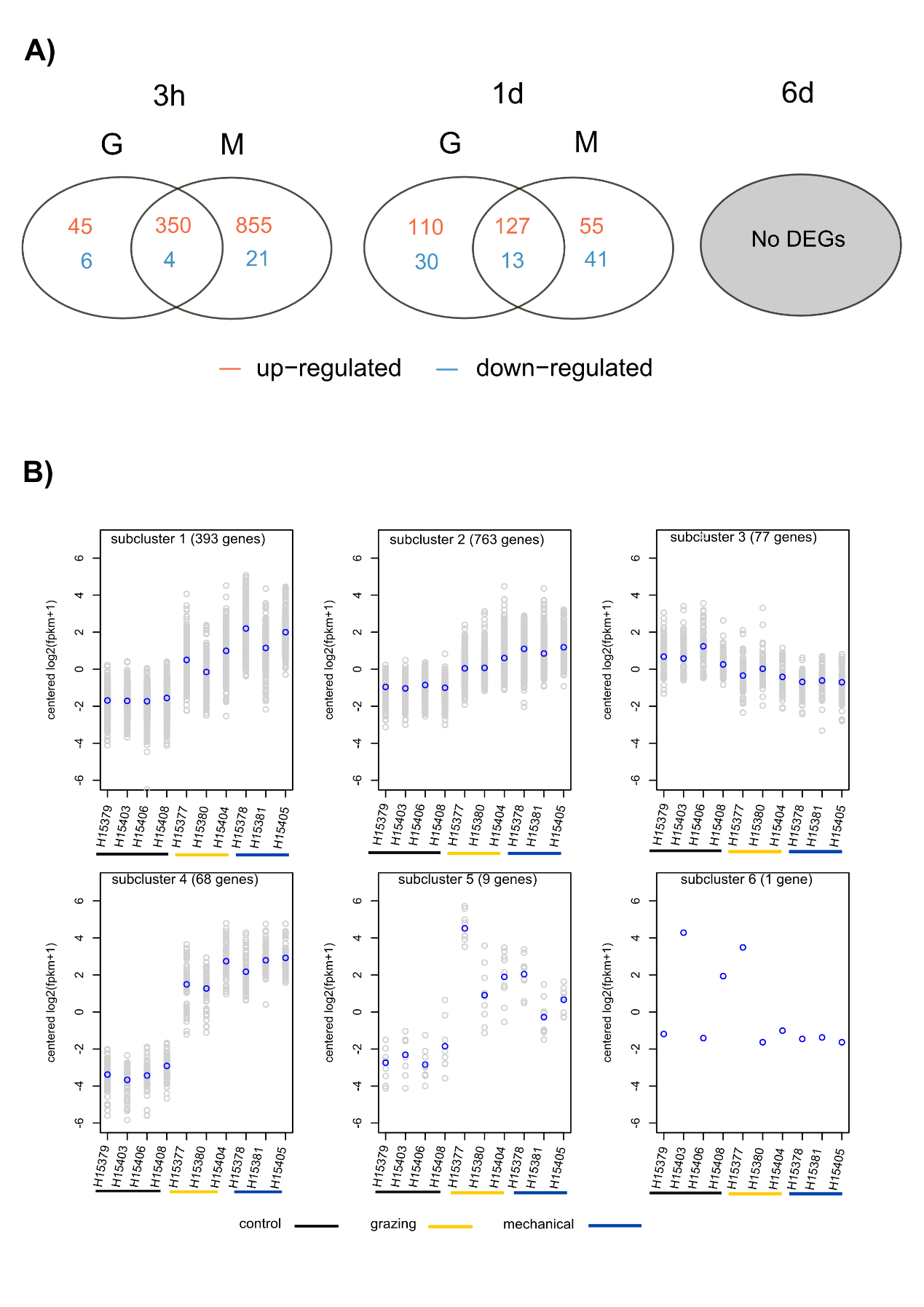
**

**Supplementary Figure S3. Gene expression patterns. A)** Number of differentially expressed genes (DEGs) in grazing (G) and mechanical damage (M) groups compared to control group at different time points (3h, 1d, and 6d). Note that none differentially-expressed gene was detected 6d post-treatment. **B)** Gene clusters at 3h according to expression patterns based on the hierarchically clustered gene tree showed in the heatmap. Clusters were defined at 60% height. DEGs were defined according to FDR p-value < 0.005 and log_2_ |FC| ≥ 2 expression, as calculated in edgeR.

**
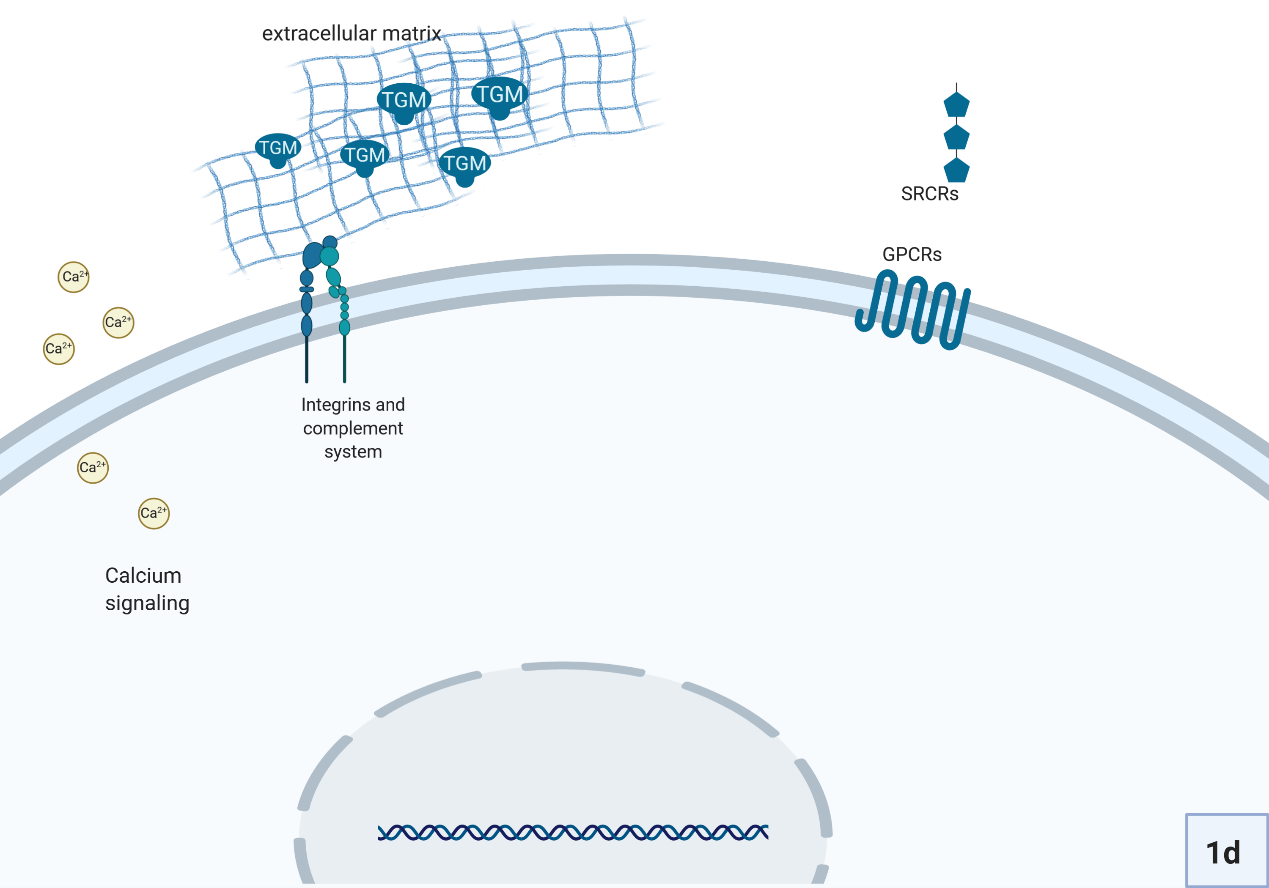
**

**Supplementary Figure S4. Suggested reconstruction of the sponge response to wounding at 1d**. Schematic representation based on the set of annotated differentially-expressed genes. The localization of the proteins and their interactions are proposed according to literature or annotation of transmembrane domains. TGM: transglutaminases. Created with BioRender.com

**
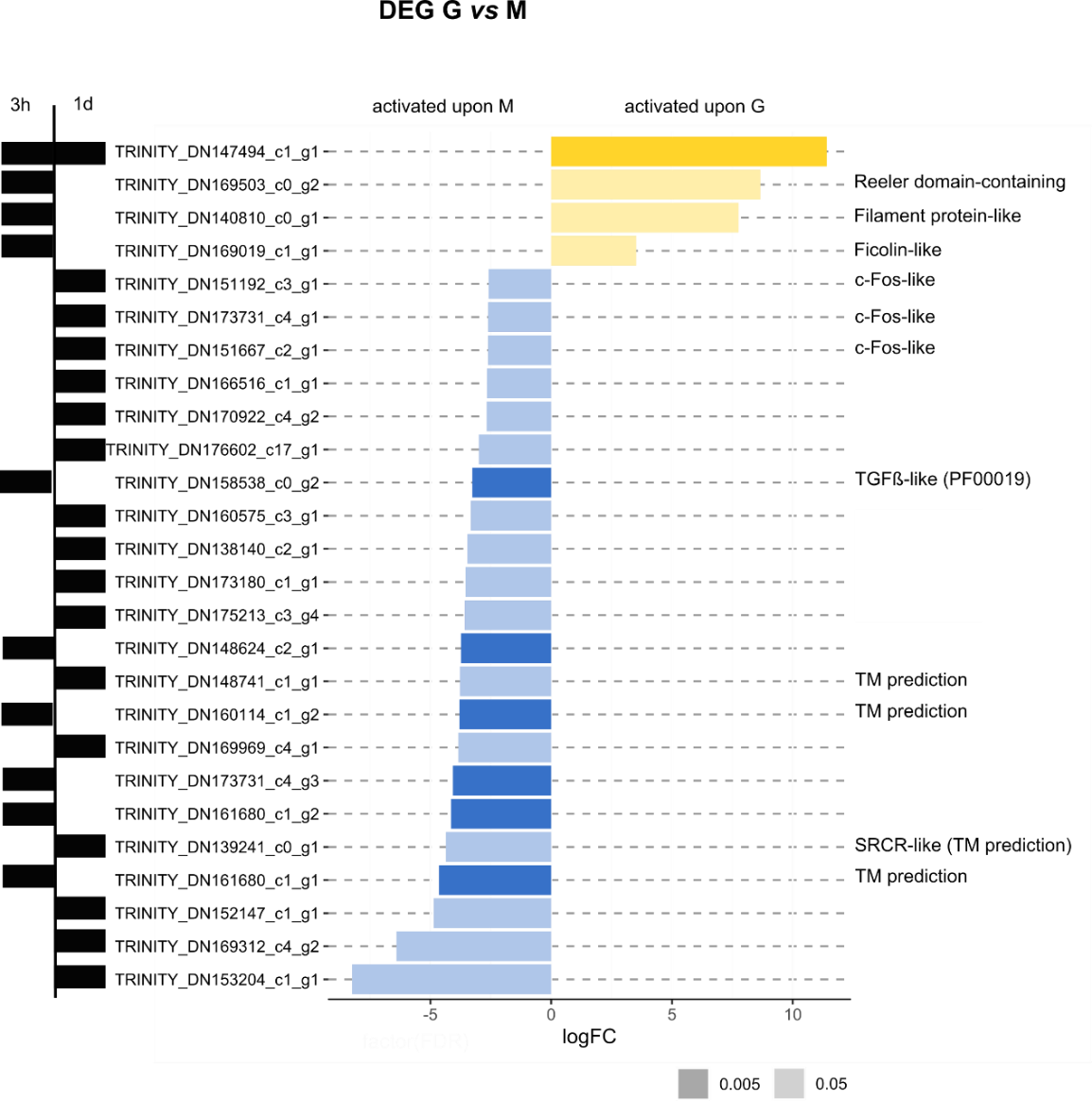
**

**Supplementary Figure S5. Direct comparison of mechanical damage to grazing response.** The direct comparison of grazing to the mechanical treatment at each experiment within 3h and 1d experiments (black bars on the left side) revealed only 7 differentially-expressed genes (DEGs) at the most restrictive definition of FDR p-value <0.005 (dark bars). When we relaxed our definition of DEG between grazing and mechanical damage from FDR p-value <0.005 to FDR p-value <0.05, we detected 19 additional DEGs (light bars). Gene names and annotation are reported. Further details on the annotation can be found in **Table S3**. Differential gene expression analyses were performed in edgeR following Trinity pipeline (Haas et al., 2013) and annotated following Trinotate pipeline (Bryant et al., 2017), unless specified otherwise. The plot was created in R with the package ggplot2. Final layout was edited in Inkscape. logFC: log_2_(fold change); TM prediction: transmembrane domain predicted. Legend refers to FDR p-value threshold.

**References**

Bryant, D. M., Johnson, K., DiTommaso, T., Tickle, T., Couger, M. B., Payzin-Dogru, D., et al. (2017). A tissue-mapped axolotl *de novo* transcriptome enables identification of limb regeneration factors. *Cell Rep.* 18, 762–776. doi:10.1016/j.celrep.2016.12.063.

Haas, B. J., Papanicolaou, A., Yassour, M., Grabherr, M., Philip, D., Bowden, J., et al. (2013). De novo transcript sequence reconstruction from RNA-Seq: reference generation and analysis with Trinity. *Nat. Protoc.* 8, 1494. doi:10.1038/nprot.2013.084.De.

Menzel, P., and Krogh, A. (2015). Fast and sensitive taxonomic classification for metagenomics with Kaiju. *Nat. Commun.* 7, 11257. doi:10.1101/031229.

Szklarczyk, D., Gable, A. L., Lyon, D., Junge, A., Wyder, S., Huerta-Cepas, J., et al. (2019). STRING v11: protein–protein association networks with increased coverage, supporting functional discovery in genome-wide experimental datasets. *Nucleic Acids Res.* 47, D607–D613. doi:10.1093/nar/gky1131.

Wu, Y.-C., García-Altares, M., Pintó, B., Ribes, M., Hentschel, U., and Pita, L. (2020). Opisthobranch grazing results in mobilisation of spherulous cells and re-allocation of secondary metabolites in the sponge *Aplysina aerophoba*. *Sci. Rep.* 10, 21934. doi:10.1038/s41598-020-78667-7.
